# Cost-Utility Analysis of the EVOLVO Study on Remote Monitoring for Heart Failure Patients With Implantable Defibrillators: Randomized Controlled Trial

**DOI:** 10.2196/jmir.2587

**Published:** 2013-05-30

**Authors:** Paolo Zanaboni, Maurizio Landolina, Maurizio Marzegalli, Maurizio Lunati, Giovanni B Perego, Giuseppe Guenzati, Antonio Curnis, Sergio Valsecchi, Francesca Borghetti, Gabriella Borghi, Cristina Masella

**Affiliations:** ^1^Norwegian Centre for Integrated Care and TelemedicineUniversity Hospital of North NorwayTromsøNorway; ^2^Cardiology DepartmentFondazione Policlinico S Matteo IRCCSPaviaItaly; ^3^Cardiology DepartmentS Carlo Borromeo HospitalMilanItaly; ^4^Cardiovascular DepartmentNiguarda HospitalMilanItaly; ^5^Cardiology DivisionIstituto Auxologico S Luca HospitalMilanItaly; ^6^Cardiology DepartmentS. Carlo Borromeo HospitalMilanItaly; ^7^Cardiology UnitOspedali CiviliBresciaItaly; ^8^Clinical DepartmentMedtronic ItaliaRomeItaly; ^9^CEFRIELRegione LombardiaMilanItaly; ^10^Department of Management, Economics and Industrial EngineeringPolitecnico di MilanoMilanItaly

**Keywords:** telemedicine, heart failure, implantable defibrillators, cost-effectiveness

## Abstract

**Background:**

Heart failure patients with implantable defibrillators place a significant burden on health care systems. Remote monitoring allows assessment of device function and heart failure parameters, and may represent a safe, effective, and cost-saving method compared to conventional in-office follow-up.

**Objective:**

We hypothesized that remote device monitoring represents a cost-effective approach. This paper summarizes the economic evaluation of the Evolution of Management Strategies of Heart Failure Patients With Implantable Defibrillators (EVOLVO) study, a multicenter clinical trial aimed at measuring the benefits of remote monitoring for heart failure patients with implantable defibrillators.

**Methods:**

Two hundred patients implanted with a wireless transmission–enabled implantable defibrillator were randomized to receive either remote monitoring or the conventional method of in-person evaluations. Patients were followed for 16 months with a protocol of scheduled in-office and remote follow-ups. The economic evaluation of the intervention was conducted from the perspectives of the health care system and the patient. A cost-utility analysis was performed to measure whether the intervention was cost-effective in terms of cost per quality-adjusted life year (QALY) gained.

**Results:**

Overall, remote monitoring did not show significant annual cost savings for the health care system (€1962.78 versus €2130.01; *P*=.80). There was a significant reduction of the annual cost for the patients in the remote arm in comparison to the standard arm (€291.36 versus €381.34; *P*=.01). Cost-utility analysis was performed for 180 patients for whom QALYs were available. The patients in the remote arm gained 0.065 QALYs more than those in the standard arm over 16 months, with a cost savings of €888.10 per patient. Results from the cost-utility analysis of the EVOLVO study show that remote monitoring is a cost-effective and dominant solution.

**Conclusions:**

Remote management of heart failure patients with implantable defibrillators appears to be cost-effective compared to the conventional method of in-person evaluations.

**Trial Registration:**

ClinicalTrials.gov NCT00873899; http://clinicaltrials.gov/show/NCT00873899 (Archived by WebCite at http://www.webcitation.org/6H0BOA29f).

## Introduction

Recent guidelines based on the evidence from randomized controlled trials recommend the use of implantable cardioverter defibrillators (ICD) and defibrillators for cardiac resynchronization therapy (CRT-D) for the management of chronic heart failure (HF) patients [1]. The conventional approach to cardiac device follow-up consists of scheduled in-office visits at intervals ranging from 3 to 6 months [2]. Because of increasing patient volumes, routine follow-up contributes a significant burden to already overstrained clinics in terms of time, capital, and human resources required, and to patients and caregivers in terms of travel and time. Remote monitoring allows assessment of device function [3] and patients’ HF-related parameters at home [4], and may represent a safe, effective, and cost-saving way to significantly reduce in-office follow-up visits that are a burden for both hospitals and patients [5].

Cost-effectiveness and cost-utility analyses are scientific approaches that can help justify the value of new interventions and, thus, informs both medical decision making and public policy [6]. Remote monitoring programs for HF have shown a positive effect on clinical outcomes [7]. However, the evidence for cost-effectiveness is limited and does not include the full range of perspectives [8]. In 2002, a systematic review of cost-effectiveness studies of telemedicine interventions concluded that there was no good evidence that telemedicine is a cost-effective means of delivering health care, but none of the studies used cost-utility [9]. In a recent systematic review of 47 economic evaluations of telemedicine interventions from 2004 to 2010, 11 were cost-effective analyses and 7 were cost-utility analyses [10]. In a meta-analysis of 14 randomized clinical trials of remote monitoring for patients with HF, only 4 studies using structured telephone support examined health care costs [11].

Thus, prospective health-economic studies are needed to correctly determine the clinical and economic benefits of systematic remote monitoring in patients with ICD and CRT-D [5,12]. We conducted a multicenter clinical trial, the Evolution of Management Strategies of Heart Failure Patients With Implantable Defibrillators (EVOLVO) study (ClinicalTrials.gov NCT00873899), aimed at measuring the benefits of remote monitoring of chronic HF patients implanted with wireless transmission-enabled ICD/CRT-D endowed with specific diagnostic features for HF [13]. The primary clinical endpoint of the EVOLVO study was to determine whether remote monitoring was associated with different rates of emergency department (ED) and urgent in-office visits for HF, arrhythmias, or ICD-related events compared to patients in the standard-treatment arm. Details of primary and secondary clinical endpoints are published elsewhere [14]. This paper focuses on the economic evaluation of the intervention and its cost utility. We hypothesized that remote device monitoring represents a cost-effective approach.

## Methods

### Study Design

**Figure 1:**
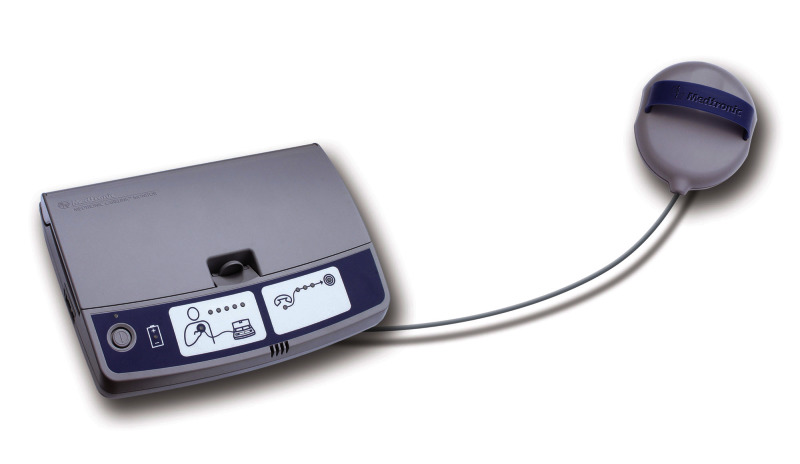
Medtronic CareLink home monitor.

**Figure 2:**
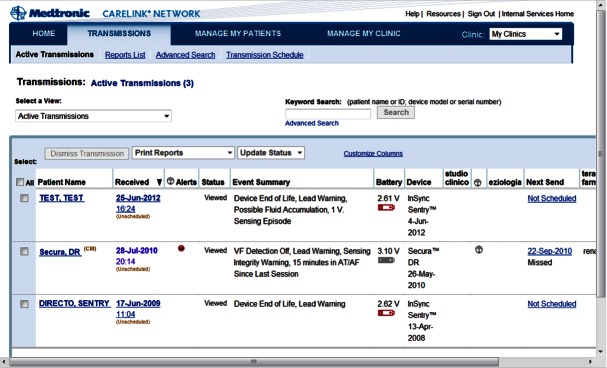
Medtronic Carelink Network.

The study design is described in detail elsewhere [13]. Briefly, the EVOLVO study is a prospective, randomized, open, multicenter clinical trial designed to compare remote monitoring of chronic HF patients with ICD/CRT-D (remote arm) to the current standard of care (standard arm; ClinicalTrials.gov NCT00873899). Two hundred patients implanted with a Medtronic (Minneapolis, MN, USA) wireless transmission-enabled ICD/CRT-D were enrolled by 6 Italian hospitals and randomized to receive either the Medtronic CareLink Home Monitor for remote transmission [15] (Figure 1) or the conventional method of in-person evaluations. Patients in the standard arm were followed for a 16-month period with scheduled in-office visits at 4, 8, 12, and 16 months. For the remote arm, patients had in-office visits at 8 and 16 months, but remote transmissions replaced their in-office visits at 4 and 12 months. In the remote arm, all alerts regarding clinical management (intrathoracic impedance for fluid accumulation monitoring, atrial arrhythmias, and ICD shocks delivered) were turned on for wireless notification through the CareLink Home Monitor, but no audible alerts were used. Hospital staff accessed patients’ data via the Web-based Medtronic CareLink Network (Figure 2). In the standard arm, patients did not have access to the CareLink Network, and the alerts were turned on for audible notification only. All system-integrity alerts were turned on for both wireless and audible notification in the remote arm and for only audible notification in the standard arm. Management strategies and data collection were predefined and have been previously described [13].

The research protocol of this study was approved by the Institutional Review Boards of the 6 participating hospitals (4 hospitals in Milan, 1 in Pavia, and 1 in Brescia). The investigation conforms to the principles outlined in the Declaration of Helsinki. All patients gave written informed consent. This trial is reported in accordance with the Consolidated Standards of Reporting Trials of Electronic and Mobile Health Applications and Online Telehealth (CONSORT-EHEALTH) [16].

### Objectives

This paper summarizes the economic evaluation of the intervention. The analysis was conducted with the perspectives of the health care system and the patient. A cost-utility analysis was performed to measure whether the intervention was cost-effective in terms of cost per quality-adjusted life year (QALY) gained.

### Health Care System Perspective

The objective of this analysis was to determine whether the total costs incurred by the health care authority for the patients in the remote arm were different from those in the standard arm. Costs included urgent and nonurgent in-office visits, scheduled and unscheduled remote follow-ups, ED visits, hospitalizations, and diagnostic examinations. All costs are expressed in Euro (€) and refer to the fiscal year 2010. A top-down approach was used for in-office visits, ED visits, hospitalizations, and diagnostic examinations. These costs, therefore, correspond to the specific public tariffs from the diagnosis-related group (DRG) system offered by the regional health care authority. Remote follow-ups were not covered by an official reimbursement scheme in Italy at the time this study was conducted, as in other European countries [17]. However, the attribution of an economic value to remote follow-ups was necessary to properly include their cost for the health care system and to compare remote to standard management. In the countries where a reimbursement for remote follow-ups is recognized, this is in-line with that of in-office visits [15,18,19]. Thus, the cost of a remote follow-up was assumed to be equal to that of an in-office visit, and reduced by 50% if not followed with a phone call to the patient. In Italy and other countries, the use of the technology (the remote monitoring device, network server, and website) is included in the initial cost of the ICD without any adjunctive fee [20], thus it does not represent a marginal cost in the economic evaluation.

However, the introduction of a yearly fee to device manufacturers covering the use of technology is a current topic of debate [21]. France is the only European country where the service is covered by the national health care insurance, with an average fee of €900 for the life of the device [22]. Therefore, a different scenario was included assuming a fee of €900 per patient for renting the remote monitoring device, the network server, and the website. An average device life span of 5 years was assumed according to recent studies [23,24]. In the EVOLVO study, the patients in both study arms were implanted with the same devices (ie, remote monitoring capabilities were available for both the intervention arm and the control arm). Therefore, the cost of the device was not included in the economic evaluation because it did not represent a marginal cost. All Medtronic ICDs currently sold in the Italian market have remote monitoring capabilities, as do most ICDs sold in Italy. Moreover, the tariff received by the hospitals for an ICD implantation is fixed and it does not vary according to the type of device implanted. New hardware investments were not requested for remote monitoring in the hospitals because the existent information technology services of the hospitals were sufficient to support the transmissions. Transportation costs were included in the patient perspective only because they are not reimbursed by the health care system. Data regarding the number of activities provided and events occurring to patients during the study period were systematically collected in an electronic database.

### Patient Perspective

The objective of this analysis was to determine whether the costs for in-office and ED visits incurred by patients and their caregivers in the remote arm were different from those in the standard arm. The cost of each visit was related to out-of-pocket expenses, including transportation, room and board, and wages lost by patients and family caregivers. Because the newest generation of Medtronic devices are able to automatically transmit data wirelessly, the time used by patients for remote follow-ups was null. These data were collected through questionnaires administered to patients at baseline. Fares of €0.47 per kilometer and €25.67 per hour were used for travel by car and by taxi, respectively. Patient-reported costs were used for other means of transportation and room and board. National wage data were used to calculate wages lost by patients and family caregivers. Hourly wages of €7.82, €10.19, €18.99, €15.06, and €10.40 were used for workers, employees, managers, entrepreneurs, and other self-employed workers, respectively.

### Cost-Utility Analysis

A cost-utility analysis was conducted using the costs assessed with the health care system perspective and QALYs. QALYs were calculated based on the answers of the EQ-5D questionnaires submitted by each patient at baseline and at 16 months. Utility values (from 0 to 1) were calculated using the European EQ-net VAS set [25]. Utility values were calculated only if all 5 of the EQ-5D dimensions were answered. Moreover, missing utility values at the study exit were imputed using regression models [26], in which the dependent variable was the utility value at 16 months, and the independent variable was the baseline value. Finally, the cost-utility ratio was computed as differential costs between remote arm and standard arm over 16 months, and differential QALYs. Because of the presence of a baseline imbalance in mean utility values between the study arms, a regression-based adjustment was applied to calculate differential QALYs controlling for baseline utility values [27].

### Statistical Analysis

Descriptive statistics were reported as mean and standard deviation (SD) for normally distributed continuous variables or median and interquartile range (IQR) in the case of skewed distributions. Normality of distribution of statistics was tested by means of the nonparametric Kolmogorov–Smirnov test. Differences between mean data were compared by using *t* tests. A Mann–Whitney nonparametric test was used to compare non-Gaussian variables. Differences in proportions were compared by using chi-square (χ^2^) tests.

Cost data are typically highly skewed because a few patients incur particularly high costs. Despite the usual skewness in the distribution of costs, statistical analysis comparing medians and using standard nonparametric methods may provide misleading conclusions [28]. The arithmetic mean is the most informative measure for policy decisions, and the *t* test on untransformed data is appropriate for costs because it is the only method addressing a comparison of arithmetic means [29]. Moreover, the *t* test is considered reliable for moderately large sample sizes. Therefore, the *t* test was used for cost analyses. A *P* value <.05 was considered significant. All statistical analyses were performed by using IBM SPSS Statistics version 19 (IBM SPSS, New York, NY, USA).

## Results

### Baseline Characteristics and Summary of Clinical Endpoints

**Figure 3:**
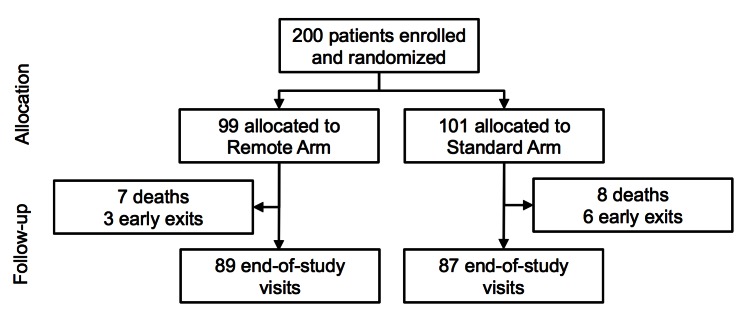
Consolidated Standards of Reporting Trials (CONSORT) diagram of the study.

Table 1 summarizes the baseline characteristics of patients. Ninety-nine patients were randomly assigned to the remote arm and 101 patients to the standard arm. Demographic and clinical parameters were similar between the study arms. Fifteen patients died during the course of the study (7 in the remote arm and 8 in the standard arm), and 9 patients were withdrawn (3 patients in the remote arm and 6 in the standard arm) (Figure 3). Table 2 summarizes the results of the primary and secondary clinical endpoints, expressed as number of events and annualized rates per patient-year. A detailed analysis of the baseline characteristics of patients and the clinical endpoints is published elsewhere [14].

### Health Care System Costs

The cost of in-office visits was €23.75, the mean cost of ED visits was €28.91 (range €22.38-€29.69), the mean cost of hospitalizations was €3865.45 (range €213.61-€25,727.70), and the mean cost of diagnostic examinations was €17.71 (range €3.95-€102.93). The mean annual cost for the management of the patients in the remote arm was lower than that in the standard arm (€1962.78 versus €2130.01; *P*=.80), although statistical significance was not reached (Table 3). Overall, remote monitoring of HF patients with implantable defibrillators did not show significant annual cost savings for the health care system. Focusing on the cost components, remote monitoring implied, on average, a lower cost for protocol-defined clinic visits than the standard management, since 2 of 4 in-office visits were replaced by scheduled remote follow-ups. According to the primary clinical endpoint, the cost of ED visits and urgent in-office visits was statistically significantly lower in the remote arm (*P*=.04). But remote monitoring required higher costs for nonurgent in-office visits, and an additional cost to perform unscheduled remote follow-ups as a consequence of automatic wireless remote notifications via CareLink. Most of the annual cost savings (€223.80) were from hospitalizations, which represents the main cost component (91% of the annual cost in the standard arm). Specifically, some of the patients in the standard arm experienced a higher number of HF-related hospitalizations compared to the patients in the remote arm. However, no statistically significant difference was detected between the 2 groups. Finally, the cost of diagnostic examinations was similar between the 2 groups.

**Table 1:** Demographics and baseline clinical characteristics of patients at the time of enrollment (N=200).

Patient characteristics		Standard arm (n=101)	Remote arm (n=99)	*P* value
Male gender, n (%)		76 (75.2)	81 (81.9)	.34
Age (years), median (IQR)		69 (60-73)	66 (60-72)	.14
**New York Heart Association (NYHA) class, n (%)**				.80
	Class I	13 (12.9)	11 (11.1)	
	Class II	68 (67.3)	71 (71.7)	
	Class III	20 (19.8)	17 (17.2)	
Primary prevention, n (%)		95 (94.1)	87 (87.9)	.20
Time since implantation >6 months, n (%)		46 (45.5)	45 (45.5)	.90
**Comorbidities, n (%)**				
	Hypertension	52 (51.5)	46 (46.5)	.57
	Diabetes	26 (25.7)	22 (22.2)	.68
	Chronic kidney disease	22 (21.8)	21 (21.2)	1.00
	COPD	15 (14.9)	19 (19.2)	.50
LV ejection fraction (%), median (IQR)		30 (25-34)	31 (25-35)	.39

**Table 2:** Summary of the clinical endpoints expressed as number of events (annualized rate per patient-year).

Clinical endpoints		Events, n (annualized rate per patient-year)		*P* value
		Standard arm (n=101)	Remote arm (n=99)	
**Primary endpoint**				
	ED/urgent in-office visits for HF, arrhythmias, or ICD-related events	117 (0.93)	75 (0.59)	.005
**Secondary endpoints**				
	ED/urgent in-office visits for HF	92 (0.73)	48 (0.38)	<.001
	ED/urgent in-office visits for arrhythmias or ICD-related events	25 (0.20)	27 (0.21)	.65
	Total health care utilizations	726 (5.76)	559 (4.40)	<.001

**Table 3:** Comparison of health care system costs.

Health care system costs	Costs (€), mean (SD)		Mean difference (95% CI)	*P* value
	Standard arm (n=101)	Remote arm (n=99)		
Protocol-defined clinic visits	90.29 (38.58)	56.63 (38.64)	33.66 (22.89, 44.43)	<.001
ED visits and urgent in-office visits	23.60 (33.68)	14.80 (24.71)	8.81 (0.56, 17.06)	.04
Nonurgent in-office visits	20.13 (38.71)	30.81 (72.13)	–10.68 (–26.78, 5.42)	.19
Scheduled remote follow-ups	0.00 (0.00)	32.50 (9.20)	–32.50 (–34.34, –30.67)	<.001
Unscheduled remote follow-ups	0.00 (0.00)	56.42 (58.95)	–56.42 (–68.18, 44.67)	<.001
Hospitalizations	1945.82 (5247.62)	1722.02 (4106.00)	223.80 (–1091.83, 1539.44)	.74
Diagnostic examinations	50.16 (73.23)	49.60 (77.80)	0.56 (–20.50, 21.63)	.96
Mean annual cost per patient	2130.01 (5251.33)	1962.78 (4185.61)	167.23 (–1158.61, 1493.06)	.80

### Patient Costs

The mean cost of in-hospital visits was €68.37 (range €0-€1720). Results summarized in Table 4 show a statistically significant reduction of the annual cost for the patients in the remote arm in comparison to that in the standard arm (€291.36 versus €381.34; *P*=.01). Remote monitoring of HF patients with implantable defibrillators, therefore, implied cost savings to patients of 24% of their total annual cost. In particular, cost savings are because of a reduction in the number of protocol-defined clinic visits, replaced by remote follow-ups for the patients in the remote arm, together with a reduction of ED visits and urgent in-office visits.

### Cost-Utility

The EQ-5D scores at baseline and 16 months were complete for 144 patients. Fifteen patients died during the study period; therefore, a 0 utility value was assigned at the study exit. By using imputed utility missing values for 21 patients with incomplete data at 16 months, QALYs were available for 180 patients, 91 in the standard arm and 89 in the remote arm. Cost data were available for all those patients. Results from the cost-utility analysis are summarized in Table 5.

Mean utility values at baseline were slightly imbalanced between the remote arm and standard arm, but not significantly different (0.793 versus 0.737; *P*=.08). Controlling for baseline, there was a differential QALY of 0.065 between the study arms (1.032 versus 0.966; *P*=.03). The mean cost for the patients in the remote arm was lower than that in the standard arm (€2074.70 versus €2962.80; *P*=.33), although statistical significance was not reached. Therefore, patients in the remote arm gained 0.065 QALYs more than those in the standard arm, with a cost savings of €888.10 per patient over the 16-month study period. The cost-utility ratio was negative. As a consequence, the cost-utility analysis showed that remote monitoring is cost-effective compared to the conventional follow-up, representing a dominant solution. Assuming a fee of €900 per patient for using the technology over 5 years, the mean cost for the patients in the remote arm was still lower than that in the standard arm (€2304.95 versus €2962.80), with a negative cost-utility ratio.

**Table 4:** Comparison of patient costs.

Patient costs	Costs (€), mean (SD)		Mean difference (95% CI)	*P* value
	Standard arm (n=101)	Remote arm (n=99)		
Protocol-defined clinic visits	259.91 (111.07)	163.01 (111.23)	96.90 (65.90, 127.90)	<.001
ED visits and urgent in-office visits	63.48 (87.29)	39.68 (66.31)	23.81 (2.16, 45.45)	.03
Nonurgent in-office visits	57.93 (111.44)	88.67 (207.63)	–30.74 (–77.08, 15.60)	.19
Mean annual cost per patient	381.34 (202.98)	291.36 (305.53)	89.97 (17.78, 162.17)	.01

**Table 5:** Utility values, quality-adjusted life years (QALYs), and cost per patient over the 16-month study period.

Cost-utility variables	Value, mean (SD)		Mean difference (95% CI)	*P* value
	Standard arm (n=91)	Remote arm (n=89)		
Mean utility value at baseline	0.737 (0.234)	0.793 (0.179)	–0.055 (–0.117, 0.006)	.08
Mean utility value at 16 months	0.711 (0.305)	0.754 (0.275)	–0.043 (–0.128, 0.043)	.32
QALYs (controlling for baseline)	0.966 (0.231)	1.032 (0.177)	–0.066 (–0.126, –0.005)	.03
Mean cost per patient (€)	2962.80 (7323.93)	2074.70 (4581.30)	–888.10 (–906.75, 2682.95)	.33

## Discussion

### Principal Findings

The results from the cost-utility analysis of the EVOLVO study show that chronic HF patients wearing ICD/CRT-D followed with remote monitoring gained 0.065 QALYs more than those in the standard arm over the 16-month study period, with a cost savings of €888.10 per patient. Remote monitoring, therefore, appears to be a cost-effective and dominant solution compared to conventional in-office follow-up. The cost-effectiveness ratio remains negative even including a fee for the use of technology in the analysis, currently adopted only in France. These results are in-line with a meta-analysis in which cost savings from remote monitoring in HF in comparison to usual care ranged from €300 to €1000, with a QALY gain of 0.06 [30]. Results from cost-utility analyses have clear implications to inform policy makers and payers. Cost per QALY of new health interventions are often grouped in league tables, in which interventions at the top should take priority. Decisions regarding implementation can then be based on threshold values for the cost per QALY, which represents the willingness of society to pay for additional QALYs. For instance, the National Institute for Clinical Excellence (NICE) has set a range of acceptable cost-effectiveness from £20,000 to £30,000 per QALY [31], and a US $50,000 per QALY threshold has been widely used in the United States for renal dialysis [5,32]. Remote monitoring of HF patients with implantable defibrillators could be taken into consideration for large-scale implementation.

Mean costs for the health care system provide another informative measure for policy decisions and confirm that the remote device monitoring might become an institutionalized service [33]. Our analysis showed that the mean annual cost for the management of the patients in the remote arm was €167.23 lower than that in the standard arm (€1962.78 versus €2130.01; *P*=.80). The cost of scheduled and unscheduled remote follow-ups, assuming a hypothetical tariff in-line with that of in-office visits, accounted for €68.92. Therefore, according to the specific results from the EVOLVO study, the maximum value that could be allocated by the health care authority to remote monitoring of HF patients implanted with ICD/CRT-D without increasing the total budget is €256.15 per patient per year. In the Clinical Evaluation of Remote Notification to Reduce Time to Clinical Decision (CONNECT) trial, the estimated mean cost per hospitalization was significantly lower because of the shorter hospital length of stay for the remote arm. However, more detailed cost data were not collected [34]. The EVOLVO study confirms that remote monitoring implies major cost savings for hospitalizations, ED visits, and urgent in-office visits, which balance the additional cost to perform unscheduled remote follow-ups as a consequence of automatic wireless remote notifications. Moreover, as compared with standard management, remote monitoring increases the rate of appropriate in-hospital visits for clinically relevant device alerts, allows early detection of worsening symptoms [35], and decreases the time from the alert condition to the data review [14].

Implications for patients are positive and confirm the findings from previous studies. Remote monitoring has been demonstrated to be highly accepted and time saving for patients with ICD [20]. Transportation costs are a major component of the overall costs of follow-up, and the potential savings have been previously estimated [36]. The EVOLVO study provides new evidence of the economic benefits for patients and caregivers. The automatic data transmission eliminates the cost normally incurred to attend in-office visits. In our clinical protocol, 2 of 4 in-office visits were replaced by remote transmissions, with consequent savings. Additional benefits would clearly emerge if a higher number of in-office visits were replaced by remote follow-ups.

### Limitations

We acknowledge 2 methodological limitations in the economic evaluation of the EVOLVO study. First, to include the cost of remote device monitoring in the absence of a reimbursement scheme, we assumed the cost of a remote follow-up based on the tariff of an in-office visit. In a European survey, 82% of the hospitals had no established reimbursement mechanism for remote follow-up. For cases in which reimbursement was present, this was established as a tariff per visit, an annual fee per patient, or charged as a service by private companies [20]. In a Finnish study, the cost of a routine follow-up, including clinical and device evaluation by a cardiologist, was €210, whereas the fee per transmission evaluation was €55 [19]. In a US study, the cost of device interrogation in-office and by remote monitoring were US $86.92 and US $102.79, respectively [18]. The introduction of a reimbursement mechanism for remote ICD follow-up is currently under discussion in different Italian regions. The second limitation concerns the study design and the different management strategies for alerts in the 2 arms. The cost of protocol-defined clinic visits was lower in the intervention arm because, in this group, patients had remote transmissions replacing their in-office visits at 4 and 12 months, which are more costly than remote follow-ups. Moreover, the protocol imposed, for the standard arm, urgent visits for audible alerts.

### Conclusions

The results from the cost-utility analysis of the EVOLVO study demonstrate that remote management of chronic HF patients with implantable defibrillators appears to be a cost-effective solution compared to the conventional method of in-person evaluations. Remote monitoring also implies significant cost savings for the patients. Today, an increasing number of outpatient clinics are already implementing remote monitoring in daily practice [4]. Thus, a large-scale adoption could be supported.

The EVOLVO study summarizes the benefits of remote monitoring for a subgroup of the HF population, namely those patients with an implantable defibrillator. This is in-line with a recent Cochrane systematic literature review [7], in which implications for research include the need for cost-effectiveness and the stratification of the benefits across the HF patient population. Future research should focus on intervention intensity and economic evaluations of large-scale studies to tailor remote monitoring for HF patients with implantable defibrillators to the population’s needs and resources, to the geography of the population, and to patient preferences [7].

## Multimedia Appendix 1

CONSORT-EHEALTH checklist V1.6.2 [16].

## Abbreviations

CONSORT: Consolidated Standards of Reporting Trials
CRT-D: cardiac resynchronization therapy defibrillators
DRG: diagnosis-related group
ED: emergency department
EVOLVO: Evolution of Management Strategies of Heart Failure Patients With Implantable Defibrillators
HF: heart failure
ICD: implantable cardioverter defibrillators
NICE: National Institute for Clinical Excellence
NYHA: New York Heart Association
QALY: quality-adjusted life year
