# Supplementary material for: Cost-Utility Analysis of the EVOLVO Study on Remote Monitoring for Heart Failure Patients With Implantable Defibrillators: Randomized Controlled Trial
Source: J Med Internet Res. 2013 May 30;15(5):e106. doi: 10.2196/jmir.2587 (PMC3670725; doi:10.2196/jmir.2587)
Supplement: Supplementary file 1 [file jmir_v15i5e106_app1.pdf]

CONSORT-EHEALTH Checklist V1.6.2 Report

(based on CONSORT-EHEALTH V1.6), available at [http://tinyurl.com/consort-ehealth-v1-6].

2587

Date completed

2/20/2013 9:59:38

by

Paolo Zanaboni

Cost-Utility Analysis of Telemonitoring for Heart Failure Patients with Implantable Defibrillators: Results from the Evolution of Management Strategies of Heart Failure Patients with Implantable Defibrillators (EVOLVO) Randomized Controlled Trial

TITLE

1a-i) Identify the mode of delivery in the title

Yes. "Telemonitoring"

1a-ii) Non-web-based components or important co-interventions in title

1a-iii) Primary condition or target group in the title

Yes. "Heart Failure Patients with Implantable Defibrillators"

ABSTRACT

1b-i) Key features/functionalities/components of the intervention and comparator in the METHODS section of the ABSTRACT

Yes. "Two hundred patients implanted with a wireless transmission-enabled implantable defibrillator were randomized to receive either telemonitoring or conventional method of in-person evaluations. Patients were followed for 16-months with a protocol of scheduled in-office and remote visits."

1b-ii) Level of human involvement in the METHODS section of the ABSTRACT

1b-iii) Open vs. closed, web-based (self-assessment) vs. face-to-face assessments in the METHODS section of the ABSTRACT

1b-iv) RESULTS section in abstract must contain use data

1b-v) CONCLUSIONS/DISCUSSION in abstract for negative trials

INTRODUCTION

2a-i) Problem and the type of system/solution

Yes. "Due to increasing patient volumes, routine follow-up contributes a significant burden to already overstrained clinics in terms of time, capital, and human resources required, and to patients and caregivers in terms of travel and time. Remote device monitoring allows one to assess device function and HF-related parameters [3], and may represent a safe, effective, and cost-saving way to significantly reduce in-office follow-up visits that are a burden for both hospitals and patients [4]."

2a-ii) Scientific background, rationale: What is known about the (type of) system

Yes. "Telemonitoring programmes for HF showed a positive effect on clinical outcomes [7]. However, the evidence for cost-effectiveness is limited overall and does not consider the full range of perspectives [8]."

METHODS

3a) CONSORT: Description of trial design (such as parallel, factorial) including allocation ratio

"We conducted a multicenter clinical trial, the Evolution of Management Strategies of Heart Failure Patients With Implantable Defibrillators (EVOLVO) study (“ClinicalTrials.gov NCT00873899”), aimed at measuring the benefits of telemonitoring of chronic HF patients implanted with wireless-transmission-enabled ICD/CRT-D endowed with specific diagnostic features for HF [13]."

"This paper focuses on the economic evaluation of the intervention and its cost-utility. We hypothesized that remote device monitoring represents a cost-effective approach.

3b) CONSORT: Important changes to methods after trial commencement (such as eligibility criteria), with reasons

Yes. No important changes to methods occurred after trial commencement.

3b-i) Bug fixes, Downtimes, Content Changes

4a) CONSORT: Eligibility criteria for participants

"The study design is described in detail elsewhere [13]."

13.Marzegalli M, Landolina M, Lunati M, Perego GB, Pappone A, Guenzati G, Campana C, Frigerio M, Parati G, Curnis A, Colangelo I, Valsecchi S. Design of the evolution of management strategies of heart failure patients with implantable defibrillators (EVOLVO) study to assess the ability of remote monitoring to treat and triage patients more effectively. Trials. 2009; 10:42.

4a-i) Computer / Internet literacy

4a-ii) Open vs. closed, web-based vs. face-to-face assessments:

"The study design is described in detail elsewhere [13]."

13.Marzegalli M, Landolina M, Lunati M, Perego GB, Pappone A, Guenzati G, Campana C, Frigerio M, Parati G, Curnis A, Colangelo I, Valsecchi S. Design of the evolution of management strategies of heart failure patients with implantable defibrillators (EVOLVO) study to assess the ability of remote monitoring to treat and triage patients more effectively. Trials. 2009; 10:42.

4a-iii) Information giving during recruitment

4b) CONSORT: Settings and locations where the data were collected

"Two hundred patients implanted with a Medtronic (Minneapolis, MN) wireless transmission-enabled ICD/CRT-D were enrolled by 6 Italian hospitals and randomized to receive either the Medtronic CareLink monitor for remote transmission [15] or the conventional method of in-person evaluations."

"The study design is described in detail elsewhere [13]."

13.Marzegalli M, Landolina M, Lunati M, Perego GB, Pappone A, Guenzati G, Campana C, Frigerio M, Parati G, Curnis A, Colangelo I, Valsecchi S. Design of the evolution of management strategies of heart failure patients with implantable defibrillators (EVOLVO) study to assess the ability of remote monitoring to treat and triage patients more effectively. Trials. 2009; 10:42.

4b-i) Report if outcomes were (self-)assessed through online questionnaires

Yes. Outcomes were not based on self-assessed through online questionnaires. A baseline questionnaire was submitted to patients to calculate costs with the patient perspective.

"Data were collected through questionnaires administered to patients at baseline."

4b-ii) Report how institutional affiliations are displayed

5) CONSORT: Describe the interventions for each group with sufficient details to allow replication, including how and when they were actually administered

5-i) Mention names, credential, affiliations of the developers, sponsors, and owners

5-ii) Describe the history/development process

5-iii) Revisions and updating

5-iv) Quality assurance methods

5-v) Ensure replicability by publishing the source code, and/or providing screenshots/screen-capture video, and/or providing flowcharts of the algorithms used

5-vi) Digital preservation

5-vii) Access

"The study design is described in detail elsewhere [13]."

13.Marzegalli M, Landolina M, Lunati M, Perego GB, Pappone A, Guenzati G, Campana C, Frigerio M, Parati G, Curnis A, Colangelo I, Valsecchi S. Design of the evolution of management strategies of heart failure patients with implantable defibrillators (EVOLVO) study to assess the ability of remote monitoring to treat and triage patients more effectively. Trials. 2009; 10:42.

5-viii) Mode of delivery, features/functionalities/components of the intervention and comparator, and the theoretical framework

"The study design is described in detail elsewhere [13]."

13.Marzegalli M, Landolina M, Lunati M, Perego GB, Pappone A, Guenzati G, Campana C, Frigerio M, Parati G, Curnis A, Colangelo I, Valsecchi S. Design of the evolution of management strategies of heart failure patients with implantable defibrillators (EVOLVO) study to assess the ability of remote monitoring to treat and triage patients more effectively. Trials. 2009; 10:42.

5-ix) Describe use parameters

5-x) Clarify the level of human involvement

5-xi) Report any prompts/reminders used

Yes. No reminders were used.

5-xii) Describe any co-interventions (incl. training/support)

"The study design is described in detail elsewhere [13]."

13.Marzegalli M, Landolina M, Lunati M, Perego GB, Pappone A, Guenzati G, Campana C, Frigerio M, Parati G, Curnis A, Colangelo I, Valsecchi S. Design of the evolution of management strategies of heart failure patients with implantable defibrillators (EVOLVO) study to assess the ability of remote monitoring to treat and triage patients more effectively. Trials. 2009; 10:42.

6a) CONSORT: Completely defined pre-specified primary and secondary outcome measures, including how and when they were assessed

Yes. "This paper summarizes the economic evaluation of the intervention. The analysis was conducted with the perspectives of the health care system and the patient. A cost-utility analysis was performed to measure whether the intervention was cost-effective in terms of cost per quality adjusted life year (QALY) gained."

6a-i) Online questionnaires: describe if they were validated for online use and apply CHERRIES items to describe how the questionnaires were designed/deployed

6a-ii) Describe whether and how “use” (including intensity of use/dosage) was defined/measured/monitored

6a-iii) Describe whether, how, and when qualitative feedback from participants was obtained

6b) CONSORT: Any changes to trial outcomes after the trial commenced, with reasons

Yes. No changes to trial outcomes occurred after the trial commenced.

7a) CONSORT: How sample size was determined

7a-i) Describe whether and how expected attrition was taken into account when calculating the sample size

7b) CONSORT: When applicable, explanation of any interim analyses and stopping guidelines

"The study design is described in detail elsewhere [13]."

13.Marzegalli M, Landolina M, Lunati M, Perego GB, Pappone A, Guenzati G, Campana C, Frigerio M, Parati G, Curnis A, Colangelo I, Valsecchi S. Design of the evolution of management strategies of heart failure patients with implantable defibrillators (EVOLVO) study to assess the ability of remote monitoring to treat and triage patients more effectively. Trials. 2009; 10:42.

8a) CONSORT: Method used to generate the random allocation sequence

"The study design is described in detail elsewhere [13]."

13.Marzegalli M, Landolina M, Lunati M, Perego GB, Pappone A, Guenzati G, Campana C, Frigerio M, Parati G, Curnis A, Colangelo I, Valsecchi S. Design of the evolution of management strategies of heart failure patients with implantable defibrillators (EVOLVO) study to assess the ability of remote monitoring to treat and triage patients more effectively. Trials. 2009; 10:42.

8b) CONSORT: Type of randomisation; details of any restriction (such as blocking and block size)

"The study design is described in detail elsewhere [13]."

13.Marzegalli M, Landolina M, Lunati M, Perego GB, Pappone A, Guenzati G, Campana C, Frigerio M, Parati G, Curnis A, Colangelo I, Valsecchi S. Design of the evolution of management strategies of heart failure patients with implantable defibrillators (EVOLVO) study to assess the ability of remote monitoring to treat and triage patients more effectively. Trials. 2009; 10:42.

9) CONSORT: Mechanism used to implement the random allocation sequence (such as sequentially numbered containers), describing any steps taken to conceal the sequence until interventions were assigned

"The study design is described in detail elsewhere [13]."

13.Marzegalli M, Landolina M, Lunati M, Perego GB, Pappone A, Guenzati G, Campana C, Frigerio M, Parati G, Curnis A, Colangelo I, Valsecchi S. Design of the evolution of management strategies of heart failure patients with implantable defibrillators (EVOLVO) study to assess the ability of remote monitoring to treat and triage patients more effectively. Trials. 2009; 10:42.

10) CONSORT: Who generated the random allocation sequence, who enrolled participants, and who assigned participants to interventions

"The study design is described in detail elsewhere [13]."

13.Marzegalli M, Landolina M, Lunati M, Perego GB, Pappone A, Guenzati G, Campana C, Frigerio M, Parati G, Curnis A, Colangelo I, Valsecchi S. Design of the evolution of management strategies of heart failure patients with implantable defibrillators (EVOLVO) study to assess the ability of remote monitoring to treat and triage patients more effectively. Trials. 2009; 10:42.

11a) CONSORT: Blinding - If done, who was blinded after assignment to interventions (for example, participants, care providers, those assessing outcomes) and how

11a-i) Specify who was blinded, and who wasn't

"The study design is described in detail elsewhere [13]."

13.Marzegalli M, Landolina M, Lunati M, Perego GB, Pappone A, Guenzati G, Campana C, Frigerio M, Parati G, Curnis A, Colangelo I, Valsecchi S. Design of the evolution of management strategies of heart failure patients with implantable defibrillators (EVOLVO) study to assess the ability of remote monitoring to treat and triage patients more effectively. Trials. 2009; 10:42.

11a-ii) Discuss e.g., whether participants knew which intervention was the "intervention of interest" and which one was the "comparator"

11b) CONSORT: If relevant, description of the similarity of interventions

Not relevant for this study.

12a) CONSORT: Statistical methods used to compare groups for primary and secondary outcomes

Yes. "Cost data are typically highly skewed, because a few patients incur particularly high costs. Despite the usual skewness in the distribution of costs, statistical analysis comparing medians and using standard non-parametric methods may provide misleading conclusions [23]. The arithmetic mean is the most informative measure for policy decisions, and the t-test on untransformed data is appropriate for costs, since it is the only method addressing a comparison of arithmetic means [24]. Moreover, the t-test is considered reliable for moderately large sample sizes. The t-test was therefore performed for cost analyses. A p-value <.05 was considered significant. All statistical analyses were performed by using IBM SPSS Statistics version 19 (IBM SPSS, New York, USA)."

12a-i) Imputation techniques to deal with attrition / missing values

Yes. "Utility values were calculated only if all the five EQ-5D dimensions were answered. Moreover, missing utility values at the study exit were imputed using regression models [21], where the dependent variable was the utility value at 16 months, and the independent variable was the baseline value."

12b) CONSORT: Methods for additional analyses, such as subgroup analyses and adjusted analyses

No subgroups analyses were conducted.

RESULTS

13a) CONSORT: For each group, the numbers of participants who were randomly assigned, received intended treatment, and were analysed for the primary outcome

"A detailed analysis of the baseline characteristics of patients and the clinical endpoints is published elsewhere |14]."

14.Landolina M, Perego GB, Lunati M, Curnis A, Guenzati G, Vicentini A, Parati G, Borghi G, Zanaboni P, Valsecchi S, Marzegalli M. Remote Monitoring Reduces Healthcare Utilization and Improves Quality of Care in Heart Failure Patients with Implantable Defibrillator: The EVOLVO (Evolution of Management Strategies of Heart Failure Patients with Implantable Defibrillators) Study. Circulation. 2012; 125:2985-92.

13b) CONSORT: For each group, losses and exclusions after randomisation, together with reasons

Yes. "Fifteen patients died during the course of the study (7 in the remote arm and 8 in the standard arm), and 9 patients were withdrawn (3 patients in the remote arm and 6 in the standard arm) (Figure 1)."

13b-i) Attrition diagram

14a) CONSORT: Dates defining the periods of recruitment and follow-up

"The study design is described in detail elsewhere [13]."

13.Marzegalli M, Landolina M, Lunati M, Perego GB, Pappone A, Guenzati G, Campana C, Frigerio M, Parati G, Curnis A, Colangelo I, Valsecchi S. Design of the evolution of management strategies of heart failure patients with implantable defibrillators (EVOLVO) study to assess the ability of remote monitoring to treat and triage patients more effectively. Trials. 2009; 10:42.

14a-i) Indicate if critical "secular events" fell into the study period

14b) CONSORT: Why the trial ended or was stopped (early)

The trial did not end or was stopped early.

15) CONSORT: A table showing baseline demographic and clinical characteristics for each group

Yes. "Table 1 summarises the baseline characteristics of patients."

15-i) Report demographics associated with digital divide issues

Yes. "Table 1 summarises the baseline characteristics of patients."

16a) CONSORT: For each group, number of participants (denominator) included in each analysis and whether the analysis was by original assigned groups

16-i) Report multiple "denominators" and provide definitions

Yes.

16-ii) Primary analysis should be intent-to-treat

17a) CONSORT: For each primary and secondary outcome, results for each group, and the estimated effect size and its precision (such as 95% confidence interval)

Yes.

17a-i) Presentation of process outcomes such as metrics of use and intensity of use

17b) CONSORT: For binary outcomes, presentation of both absolute and relative effect sizes is recommended

Yes. No binary outcomes were reported.

18) CONSORT: Results of any other analyses performed, including subgroup analyses and adjusted analyses, distinguishing pre-specified from exploratory

No other analyses were performed.

18-i) Subgroup analysis of comparing only users

19) CONSORT: All important harms or unintended effects in each group

Yes. No important harms or unintended effects were present for both groups.

19-i) Include privacy breaches, technical problems

19-ii) Include qualitative feedback from participants or observations from staff/researchers

DISCUSSION

20) CONSORT: Trial limitations, addressing sources of potential bias, imprecision, multiplicity of analyses

20-i) Typical limitations in ehealth trials

Yes. "We acknowledge two methodological limitations in the economic evaluation of the EVOLVO study. First, in order to include the cost of remote device monitoring, but in the absence of a reimbursement scheme, we assumed the cost of a remote visit based on the tariff of an in-office visit. In a European survey, 82% of the hospitals had no established reimbursement mechanism for remote follow-up. Where reimbursement was present, this was established as tariff per visit, annual fee per patient, or charged as a service by private companies [19]. In a Finnish study, the cost of a routine follow-up, including clinical and device evaluation by a cardiologist, was €210, while the fee per transmission evaluation was €55 [18]. In a US study, the cost of device interrogation in-office and using telemonitoring were $86.92 and $102.79, respectively [17]. The introduction of a reimbursement mechanism for remote ICD follow-up is currently under discussion in different Italian Regions. The second limitation concerns the study design and the different management strategies for alerts in the two arms. The cost of protocol-defined clinic visits was lower in the intervention arm because, in this group, patients had remote transmissions replacing their in-office visits at 4 and 12 months, which are more costly than remote visits. Moreover, the protocol imposed, for the standard arm, urgent visits for audible alerts."

21) CONSORT: Generalisability (external validity, applicability) of the trial findings

21-i) Generalizability to other populations

21-ii) Discuss if there were elements in the RCT that would be different in a routine application setting

22) CONSORT: Interpretation consistent with results, balancing benefits and harms, and considering other relevant evidence

22-i) Restate study questions and summarize the answers suggested by the data, starting with primary outcomes and process outcomes (use)

Yes. "The main finding of the economic evaluation of the EVOLVO study is that chronic HF patients wearing ICD/CRT-D followed with telemonitoring gained 0.065 QALYs more than those in the standard arm over 16 months, with a cost savings of €888.10 per patient. Telemonitoring, therefore, appears to be a cost-effective and dominant solution over the conventional in-office follow-up. These results are in line with a meta-analysis where cost savings from telemonitoring in HF in comparison to usual care ranged from €300 to €1000, with a QALYs gain of 0.06 [25]. Results from cost-utility analyses have clear implications to inform policy makers and payers. Cost per QALY of new health interventions are often grouped in league tables, where interventions at the top should take priority. Decisions regarding implementation can then be based on threshold values for the cost per QALY, which represents the willingness of society to pay for additional QALYs. For instance, the National Institute for Clinical Excellence (NICE) has set a range of acceptable cost-effectiveness from £20,000 to £30,000 per QALY [26], and the $50,000 per QALY threshold has been widely used in the United States based on renal dialysis [5,27]. Telemonitoring of HF patients with implantable defibrillators appears to be a dominant solution which could be taken into consideration for large-scale implementation."

"Mean costs for the health care system provide another informative measure for policy decisions and confirm that the remote device monitoring might become an institutionalized service [28]. Our analysis showed that the mean annual cost for the management of the patients in the remote arm was €167.23 lower than that in the standard arm (€1962.78 versus €2130.01). The cost of scheduled and unscheduled remote visits assuming a hypothetical tariff in line with that of in-office visits accounted for €68.92. Therefore, according to the specific results from the EVOLVO study, the maximum value that could be allocated by the health care authority to telemonitoring of HF patients implanted with ICD/CRT-D without increasing the total budget is €256.15 per patient per year. In the CONNECT Trial, as a result of the shorter hospital length of stay for the remote arm, the estimated mean cost per hospitalisation was significantly lower. However, more detailed cost data were not collected [29]. The EVOLVO study confirms that telemonitoring implies major cost savings for hospitalisations, ED visits and urgent in-office visits, which balance the additional cost to perform unscheduled remote visits as a consequence of automatic wireless remote notifications. Moreover, as compared with standard management, telemonitoring increases the rate of appropriate in-hospital visits for clinically relevant device alerts and decreases the time from the alert condition to the data review [14]."

"Implications for patients are positive and confirm the findings from previous studies. Telemonitoring has been demonstrated to be highly accepted and time saving for patients with ICD [19]. Transportation costs are a major component of the overall costs of follow-up, and the potential savings have been previously estimated [30]. The EVOLVO study provides new evidence of the economic benefits for patients and caregivers. The automatic data transmission eliminates the cost normally incurred to attend in-office visits. In our clinical protocol two of four in-office visits were replaced by remote transmissions, with consequent savings. Additional benefits would clearly emerge if a higher number of in-office visits were replaced by remote visits."

22-ii) Highlight unanswered new questions, suggest future research

Other information

23) CONSORT: Registration number and name of trial registry

Yes. "Trial Registration: ClinicalTrials.gov NCT00873899".

24) CONSORT: Where the full trial protocol can be accessed, if available

"Trial Registration: ClinicalTrials.gov NCT00873899"

13.Marzegalli M, Landolina M, Lunati M, Perego GB, Pappone A, Guenzati G, Campana C, Frigerio M, Parati G, Curnis A, Colangelo I, Valsecchi S. Design of the evolution of management strategies of heart failure patients with implantable defibrillators (EVOLVO) study to assess the ability of remote monitoring to treat and triage patients more effectively. Trials. 2009; 10:42.

25) CONSORT: Sources of funding and other support (such as supply of drugs), role of funders

Yes. "The EVOLVO study was supported by Italian Ministry of Health (grant RFPS-2006-2-335243) and by Regione Lombardia. Medtronic Italia provided technical support."

X26-i) Comment on ethics committee approval

x26-ii) Outline informed consent procedures

X26-iii) Safety and security procedures

X27-i) State the relation of the study team towards the system being evaluated
